# A rare case of localized peliosis hepatis during adjuvant chemotherapy including oxaliplatin mimicking a liver metastasis of colon cancer

**DOI:** 10.1186/s40792-023-01774-w

**Published:** 2023-11-15

**Authors:** Akira Dejima, Yasuji Seyama, Daisuke Nakano, Misato Takao, Soichiro Natsume, Mikiya Takao, Sakiko Nakamori, Tatsuya Kanai, Shinichiro Horiguchi, Kazushige Kawai

**Affiliations:** 1 Department of Colorectal Surgery, Tokyo Metropolitan Cancer and Infectious Diseases Center, Komagome Hospital, 3-18-22 Honkomagome, Bunyo-Ku, Tokyo, 113-0021 Japan; 2 Department of Hepato-Biliary-Pancreatic Surgery, Tokyo Metropolitan Cancer and Infectious Diseases Center, Komagome Hospital, 3-18-22 Honkomagome, Bunyo-Ku, Tokyo, 113-0021 Japan; 3 Department of Pathology, Tokyo Metropolitan Cancer and Infectious Diseases Center, Komagome Hospital, 3-18-22 Honkomagome, Bunyo-Ku, Tokyo, 113-0021 Japan

**Keywords:** Peliosis hepatis, Metastatic liver tumor, Oxaliplatin

## Abstract

**Background:**

Oxaliplatin-based regimens are commonly used as adjuvant chemotherapy following surgery for colorectal cancer. Adverse events associated with oxaliplatin include blue liver, which is caused by sinusoidal dilation and diffuse peliosis hepatis. We report herein a case of localized peliosis hepatis closely resembling a metastatic liver tumor.

**Case presentation:**

The patient, a 50-year-old male, underwent a robotically assisted colectomy for rectosigmoid colon cancer, which was discovered when hematochezia occurred. The patient received a diagnosis of pStage IIIb and was treated with four courses of CAPOX as adjuvant chemotherapy starting at postoperative month 1. At postoperative month 4, contrast-enhanced computed tomography (CT) of the abdomen revealed a 20-mm, low-density area with heterogeneous internal structure in S6/7 of the liver. Abdominal ultrasound and gadolinium ethoxybenzyl-diethylenetriaminepentaacetic acid-enhanced magnetic resonance imaging (EOB-MRI) findings led to a diagnosis of metastatic liver tumor, for which a laparoscopic partial hepatectomy was performed. The resected lesion was a dark reddish-brown nodule with indistinct margins that appeared to be continuous with the surrounding area. Histopathological analysis revealed severe, localized dilatation of the sinusoids and congestion consistent with the gross nodule. Based on these findings, localized peliosis hepatis associated with oxaliplatin-induced sinusoidal damage was diagnosed.

**Conclusions:**

Localized peliosis hepatis associated with oxaliplatin use can be difficult to distinguish from a metastatic liver tumor on imaging studies.

## Background

When a new liver mass appears after surgery for advanced colorectal cancer, liver metastasis is usually suspected. Early detection by regular imaging studies and accurate diagnosis based on various, detailed examinations are quite important [1].

In recent years, postoperative adjuvant chemotherapy for advanced colorectal cancer has become common. In particular, the introduction of chemotherapy regimens with oxaliplatin has improved the prognosis of advanced colorectal cancer [2]. On the other hand, blue liver stemming from sinusoidal dilation is a known adverse effect of the drug, and there are anecdotal reports of diffuse peliosis hepatis associated with its use [3]. The present report describes a case of peliosis hepatis caused by oxaliplatin-induced sinusoidal damage which was difficult to differentiate from a metastatic liver tumor owing to localized lesion formation and the requirement for hepatic resection.

## Case presentation

The patient was a 50-year-old male who had undergone a robotically assisted colectomy for rectosigmoid colon cancer. Histopathological analysis confirmed the diagnosis of moderately differentiated adenocarcinoma, pT3, pN1a, pM0, pStage IIIb. Starting at postoperative month 1, the patient received four cycles of CAPOX as adjuvant chemotherapy.

**Fig. 1:**
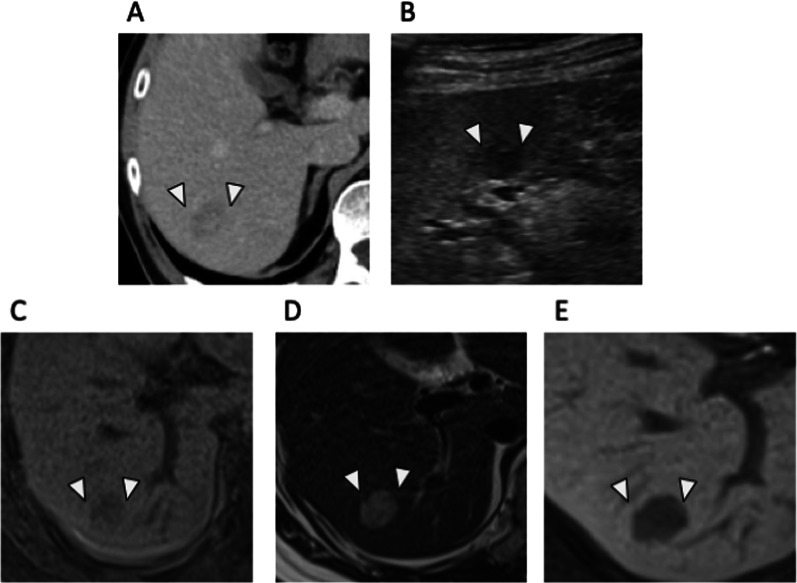
Imaging studies of the liver lesion. **A** Contrast-enhanced abdominal computed tomography demonstrating a 20-mm, low-density area in hepatic S6/7 in the portal vein phase. **B** Abdominal ultrasound showing a heterogeneous, internal mass in the hepatic S6/7. **C** EOB-MRI, low signal intensity on T1-weighted imaging. **D** EOB-MRI, high signal intensity on T2-weighted imaging. **E** EOB-MRI, low contrast uptake in EOB-hepatocyte phase

At postoperative month 4, contrast-enhanced abdominal computed tomography (CT) revealed a 20-mm, low-density area with heterogeneous internal structure in S6/7 (Fig. 1A) suggestive of a liver metastasis. On abdominal ultrasound, it was depicted as an internally heterogeneous mass (Fig. 1B). EOB-magnetic resonance imaging (MRI) demonstrated a low-signal area on T1-weighted imaging (Fig. 1C), and T2-weighted imaging demonstrated a high-signal area (Fig. 1D). The EOB hepatocellular phase demonstrated decreased contrast uptake (Fig. 1E).

Blood tests at the time of the discovery of the liver lesion revealed CEA 2.6 ng/mL, CA19-9 8.2 U/mL, AST 23 IU/L, ALT 21 IU/mL, indicating normal tumor markers and liver enzymes.

**Fig. 2:**
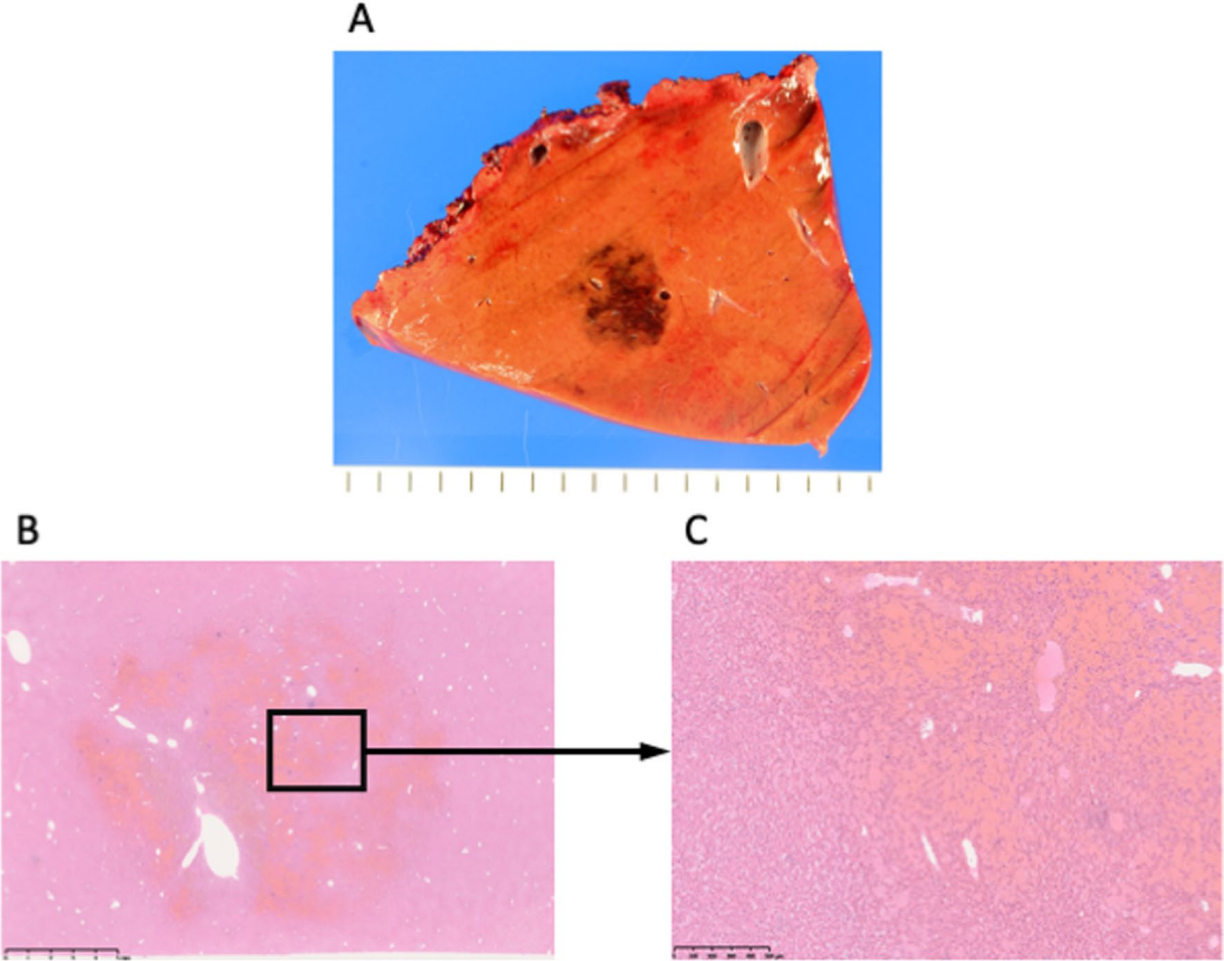
Resection sample and histological findings. **A** A dark reddish-brown nodule measuring 1.8 × 1.8 × 1.5 cm was observed in S7. The nodule had indistinct borders. **B**, **C** Localized, severe dilatation and congestion of the sinusoid consistent with the gross nodule were observed

Based on these findings, liver metastasis of colon cancer was diagnosed, and a laparoscopic partial hepatectomy was performed. Intraoperative findings revealed a solitary lesion at S7 with no obvious abnormalities in the background liver. No blue liver or splenomegaly to the varices suggestive of portal hypertension was observed intraoperatively. The resected specimen was a dark reddish-brown nodule measuring 1.8 × 1.8 × 1.5 cm with indistinct borders (Fig. 2A).

Histopathological analysis revealed severe, localized dilatation and congestion of the sinusoids consistent with the gross nodule (Fig. 2B, C). The hepatocyte cords were slightly thinned in the congested areas, but there was no obvious disorder in their arrangement, and the individual hepatocytes were not atypical. Based on these findings, peliosis hepatis caused by oxaliplatin-induced sinusoidal damage was diagnosed. The patient is now at 1.5 years post-hepatectomy and is doing well without further episodes of peliosis hepatis.

## Discussion

Peliosis hepatis was first described by Wagner in 1861 and named by Schoenlank in 1916. The first description in the English-language literature was published by Zak in 1950 [4]. Peliosis hepatis is characterized by a diffuse distribution of irregularly shaped hemorrhages (1–10 mm in diameter) within the hepatic parenchyma [5]. Although its pathogenesis is unclear, various forms of endothelial cell damage in the sinusoids lead to their dilatation, known as Disse’s cavity, which fills with blood and results in the destruction of the sinusoids [6]. The etiology of peliosis hepatis is unknown in 25–50% of cases [7], but the use of certain drugs (azathioprine, oxaliplatin, oral contraceptives, steroids), infections (tuberculosis, HIV), hematological malignancies, and renal transplantation are reportedly associated with it [8–10]. In the present case, the patient had pStage IIIb, received CAPOX as postoperative adjuvant chemotherapy, and underwent a hepatectomy after contrast-enhanced CT demonstrated a solitary mass in the liver suggestive of a metastatic liver tumor. The mass lesion demonstrated severe, localized, sinusoidal dilatation and congestion. Oxaliplatin-induced endothelial cell damage was suspected to be the cause, because blue liver caused by sinusoidal dilatation is a known adverse effect of oxaliplatin. Anecdotally, diffuse peliosis hepatis is associated with blue liver, but cases like the present one, which was difficult to distinguish from a metastatic liver tumor because of its localized formation and required a hepatic resection for a definitive diagnosis, are extremely rare [11].

There are few reports describing the findings typical of peliosis hepatis on imaging studies, such as CT and ultrasound. The characteristics of the condition are not yet well known but may include heterogeneous hypoechoic to isoechoic areas on abdominal ultrasound; low attenuation and internal heterogeneity in the late arterial and portal phases on contrast-enhanced CT [12]; low signal intensity on T1-weighted MRI; high signal intensity on T2-weighted MRI; and rich vessel presence in the arterial phase [13]. However, a differential diagnosis based on imaging studies is challenging because both peliosis hepatis and liver metastasis have varied presentations. Although the preoperative images in the present case were not typical of metastatic liver tumors, no PET–CT was performed. Peliosis hepatitis is usually isometabolic, whereas metastatic liver tumors tend to have increased uptake of 18F-FDG [14]. Therefore, PET–CT might have contributed to the differential diagnosis in the present case.

Although peliosis hepatis is definitively diagnosed on the basis of histopathological findings, the risk of bleeding is high even with ultrasound or CT-guided biopsy owing to the vascularity of the lesion, and occurrences of a life-threatening hemorrhagic shock after CT-guided biopsy have been reported [15]. While a previous study diagnosed peliosis hepatis on the basis of laparoscopic liver biopsy findings [13], the present case was preoperatively indistinguishable from a metastatic liver tumor and required a liver resection for definitive diagnosis.

Diagnosing localized, solitary peliosis hepatis developing after oxaliplatin administration, as in the present case, is difficult without a hepatectomy [16]. However, if the imaging studies of a patient who has received oxaliplatin-based chemotherapy demonstrate features that are typical of a liver metastasis, peliosis hepatis should be included in the differential diagnosis.

## Conclusions

Localized peliosis hepatis associated with oxaliplatin treatment can be difficult to distinguish from a metastatic liver tumor on imaging studies.

## Data Availability

All the data generated or analyzed during this study are included in this article.

## Abbreviations

CT: Computed tomography
EOB-MRI: Gd-EOB-PTPA-enhanced magnetic resonance imaging
